# Spatial Differentiation and Community Assembly of Soil Bacterial Communities in Permafrost Peatlands of the Greater Khingan Mountains

**DOI:** 10.3390/microorganisms14071558

**Published:** 2026-07-16

**Authors:** Shuping Kan, Zedong Liu, Dalong Ma, Weiping Yin, Xu Wang

**Affiliations:** 1 College of Geographical Sciences, Harbin Normal University, Harbin 150025, China; 2024300998@stu.hrbnu.edu.cn (S.K.); liuzedong2026@163.com (Z.L.); 2024400071@stu.hrbnu.edu.cn (W.Y.); wx18800469360@163.com (X.W.); 2 Huzhong Permafrost and Cold Environments Observation and Research Station of Heilongjiang Province, Da Hinggan Ling 165036, China; 3 Institute of Industrial Crops, Heilongjiang Academy of Agricultural Sciences, Harbin 150086, China

**Keywords:** permafrost peatlands, bacterial community, environmental drivers, spatial differentiation

## Abstract

Global warming is profoundly altering the structure and function of permafrost peatland ecosystems, but how soil microorganisms as core regulators of biogeochemical cycles respond to the process remains unclear. We investigated peatlands of the continuous, the discontinuous, and the isolated permafrost zones in the climatically sensitive high-latitude Greater Khingan Mountains using 16S rRNA gene high-throughput sequencing and soil physicochemical analysis to systematically reveal the spatial differentiation patterns, community assembly processes, and primary environmental factors of bacterial communities. The results indicated that bacterial alpha diversity was highest in the discontinuous permafrost zone, and both permafrost type and soil depth exerted significant effects on bacterial community composition. From the continuous to the isolated permafrost zones, the relative abundance of the dominant phylum Proteobacteria decreased, while phylum Chloroflexota showed a gradual increasing trend. Co-occurrence network analysis suggested that bacterial network complexity was highest in the continuous permafrost zone, and network stability decreased along the permafrost gradient. From the continuous to the isolated permafrost zone, the relative contribution of stochastic processes declined, whereas that of deterministic processes increased. Partial least squares path modeling (PLS-PM) further demonstrated that soil pH, total organic carbon (TOC), total nitrogen (TN), and soil water content (SWC) were major drivers of bacterial communities, with their effects differing among permafrost zones. Our study elucidated the synergistic evolutionary patterns of bacterial community composition, assembly mechanisms, and environmental drivers under permafrost degradation, providing key scientific evidence for predicting the feedback of high-latitude peatlands to climate warming.

## 1. Introduction

Global warming is profoundly altering the structure and function of permafrost peatland ecosystems. The low permeability of permafrost acts as an impermeable layer that impedes water infiltration while creating a persistently anoxic environment, thereby restricting plant residue decomposition and leading to its gradual accumulation into a peat layer [1,2]. Peat serves as a natural thermal insulation layer due to its extremely low thermal conductivity, thereby helping to maintain permafrost thermal stability [3]. Under the sustained regulation of this multiple sphere-coupled system, organic matter mineralization has been continuously suppressed, driving millennia of net carbon sequestration in permafrost peatlands, which have accumulated approximately 300–500 Pg of organic carbon, making them one of the massive yet vulnerable terrestrial carbon reservoirs [4,5]. However, global warming is profoundly destabilizing this carbon reservoir. High-latitude regions are getting warmer at 2–4 times the global average rate, driving widespread permafrost degradation characterized by shrinking permafrost area and a marked decline in spatial continuity, with continuous permafrost zones rapidly transitioning to discontinuous or isolated permafrost zones [6]. This process relieves the long-term suppression of organic matter decomposition by low temperatures, may drive a shift in peatlands from a historical net carbon sink to a potential net carbon source, releasing substantial amounts of greenhouse gases and creating a significant positive feedback loop to climate warming [7].

Microorganisms, serving as key drivers of material cycling in permafrost peatland ecosystems, have developed unique psychrophilic and cold-tolerant communities in frigid, oligotrophic habitats [8,9]. Bacteria are predominant in microorganisms, comprising 70–90% of the total microbial biomass in permafrost and governing the key processes of carbon and nitrogen cycling in peatlands [10]. Evidence from both laboratory incubation experiments and field in situ monitoring consistently demonstrated that permafrost thaw not only significantly reduces bacterial alpha diversity but also drives a systematic restructuring of community composition [11,12,13]. Moreover, this community shift is highly coupled with enhanced rates of soil carbon mineralization and greenhouse gas emission fluxes [14]. Studies conducted in permafrost zones of the Tibetan Plateau have revealed that along the permafrost degradation gradient, the relative abundance of copiotrophic taxa represented by Proteobacteria and Actinobacteriota declines significantly, whereas oligotrophic Acidobacteriota become markedly enriched [15]. Furthermore, active layer deepening breaks the physical barrier of permafrost, enhancing microbial dispersal connectivity along both vertical and horizontal directions [16]. The thaw-driven homogenization of habitats weakens the redox gradient at the anaerobic–aerobic interface, leading to environmental filtering and homogeneous selection gradually dominating the assembly processes of microbial communities [17]. Therefore, elucidating the response patterns, spatial heterogeneity, and key environmental controlling mechanisms of microbial communities during permafrost degradation is a critical prerequisite for predicting the evolution trajectory of peatland ecosystems in cold regions.

The Greater Khingan Mountains permafrost zone, located at the southern margin of the Eurasian high-latitude permafrost zone, covers approximately 0.39 × 10^6^ km^2^ [18]. Over the past century, the regional temperature has risen by about 1.7 °C, driving the southern boundary of permafrost northward by 50–120 km, degrading discontinuous permafrost into isolated permafrost, and causing the isolated permafrost zone to vanish completely in some areas [19]. Permafrost fragmentation and progressive thickening of the active layer have emerged as prominent features of the Greater Khingan Mountains permafrost zone, providing a unique natural experimental site for investigating the impacts of high-latitude permafrost degradation on peatland microbial communities [20]. To date, considerable progress has been made in understanding the response mechanisms of microbial communities in permafrost peatlands to global warming [21,22]. However, research has been highly concentrated in the circum-Arctic continuous permafrost zones (Alaska, western Siberia, northern Sweden) and the high-altitude permafrost zone of the Tibetan Plateau [23,24,25,26]. In contrast, the Greater Khingan Mountains permafrost zone, despite its high climatic sensitivity and pronounced permafrost degradation, has received little research attention, which has seriously constrained a comprehensive understanding of microbial biogeographic patterns at the southern edge of the high-latitude permafrost zone. Therefore, this study focused on typical peatlands of the continuous, discontinuous, and isolated permafrost zones in the Greater Khingan Mountains, collecting soil samples at three depths (0–10 cm, 10–30 cm, and 30–50 cm), and employed 16S rRNA gene high-throughput sequencing to systematically investigate the composition, diversity, and community assembly processes of bacterial communities. To our knowledge, this is the first comprehensive study of co-occurrence networks and community assembly processes of bacterial communities across three permafrost types in this region. The specific objectives of this study include: (1) revealing the differentiation patterns of bacterial community composition along permafrost types and soil depths, (2) elucidating the shifts in bacterial community assembly mechanisms across different permafrost zones, and (3) constructing of co-occurrence networks and identifying of key environmental drivers of community composition. The findings of this study will provide a scientific basis for a better understanding of microbial feedback mechanisms in peatland ecosystems to climate warming in high-latitude cold regions.

## 2. Materials and Methods

### 2.1. Study Area

The study area is located in the Greater Khingan Mountains permafrost zone (50°07′–53°33′ N, 121°10′–127°01′ E), at the southern boundary of the Eurasian high-latitude permafrost zone, and represents a typical Xing’an-Baikal regional permafrost zone [27]. Characterized by a cold temperate continental monsoon climate, this region has a snow cover period of more than 6 months, and a frost-free period of 90–120 days, with an annual mean temperature of −2.8 to −4.3 °C, and annual mean precipitation of 460–520 mm [28]. Soils are predominantly meadow soil, swamp soil, and peat soil, with a peat layer thickness of 10–90 cm and an active layer thickness of 40–200 cm. The dominant vegetation consists of *Larix gmelinii*, *Betula fruticosa*, *Vaccinium uliginosum*, *Carex appendiculata*, *Eriophorum vaginatum*, and *Sphagnum palustre*.

### 2.2. Sample Collection

Based on preliminary field surveys and systematic evaluation to exclude potential interference from external environmental factors, this study adopted a space-for-time substitution method and selected nine typical peatland plots from continuous (Mohe, Tuqiang, and Huzhong), discontinuous (Tahe, Xinlin, and Nanwenghe), and isolated (Songling, Jiagedaqi, and Dayangshu) permafrost zones in the Greater Khingan Mountains in September 2023. Three 10 m × 10 m quadrats were established in each plot, and soil samples were collected from the 0–10, 10–30, and 30–50 cm depths along an S-shaped sampling route. Samples from the same soil depth were mixed in equal quantities to form one composite sample, yielding a total of 81 samples (9 plots × 3 depths × 3 replicates). Soil samples were transported to the laboratory using sterile bags at 4 °C and after removing plant roots and stones, each sample was separated into two portions: one portion stored at −80 °C for DNA extraction and the other at 4 °C for soil physicochemical properties analysis.

### 2.3. High-Throughput Sequencing

The PowerSoil^®^ DNA Isolation Kit (MOBIO, Carlsbad, CA, USA) was applied to extract total microbial DNA, and the amplification of the bacterial 16S rRNA V3–V4 hypervariable region was performed employing the primer pair 338F/806R [29]. Once purified and quantified, the PCR products were subjected to paired-end sequencing on the Illumina MiSeq PE300 platform. Raw reads were quality-filtered using Trimmomatic (v.0.39), with a minimum average quality threshold of Q20, a 10 bp sliding window, >50 bp retained. Paired-end reads were merged using FLASH (v.1.2.11). Chimeric sequences were removed using UCHIME against SILVA database. The remaining sequences were clustered into OTUs at 97% similarity using UPARSE (v.11), and taxonomically assigned via RDP Classifier algorithm (v.2.13) with the SILVA database with a confidence threshold of 70%. Unclassified and candidate lineages are reported at the genus and phylum levels as designated by the SILVA taxonomy, a convention widely adopted in 16S rRNA amplicon-based microbial community analyses. All library sizes were rarefied to the minimum number of reads prior to calculations.

### 2.4. Soil Physicochemical Properties Analysis

Soil water content (SWC) was determined by the oven-drying method at 105 °C for 24 h. Soil pH was measured using a pH meter (LeiCi PHSJ 3F, Shanghai, China) in a 1:2.5 (*w*/*v*) soil-to-water suspension. A total organic carbon analyzer (Multi N/C 3100, Jena, Germany) was applied to quantify total organic carbon (TOC) and dissolved organic carbon (DOC) contents. The contents of ammonium nitrogen (NH_4_^+^-N) and nitrate nitrogen (NO_3_^−^-N) were determined with a continuous flow analyzer (Skalar San^++^, Breda, The Netherlands). Total nitrogen (TN) content was analyzed using the method described by Kjeldahl. The molybdenum-antimony anti-colorimetric method was used to determine total phosphorus (TP) content after H_2_SO_4_-HClO_4_ digestion.

### 2.5. Statistical Analysis

Mothur (v.1.30.2) was utilized to calculate alpha diversity indices, including Chao1, Shannon, Simpson, and Pielou_e. Principal coordinate analysis (PCoA) and permutational multivariate analysis of variance (PERMANOVA) were performed based on Bray-Curtis distances using the “vegan” package in R (v.4.1.0) [30]. Two-way ANOVA in SPSS (v.26.0) was applied to examine the impacts of permafrost type, soil depth, and their joint contribution to bacterial community diversity. The community assembly processes were quantified by the beta nearest-taxon index (βNTI) using the “picante” package in R, and the neutral community model (NCM), to determine the relative contributions of deterministic and stochastic processes. Co-occurrence networks were built on Spearman’s correlation results (|ρ| > 0.6, *p* < 0.05) utilizing the “igraph” package in R, and visualized with Gephi (v.0.9.2), a threshold commonly used in microbial network studies [31]. At a q-value filter of <0.05, all results were treated to a multiple test correction using the Benjamini-Hochberg false discovery rate (FDR). The effects of soil physicochemical properties on bacterial communities were comprehensively evaluated using Mantel tests (“ggcor” package in R), and partial least squares path modeling (PLS-PM, “plspm” package in R).

## 3. Results

### 3.1. Diversity of the Soil Bacterial Community

**Figure 1:**
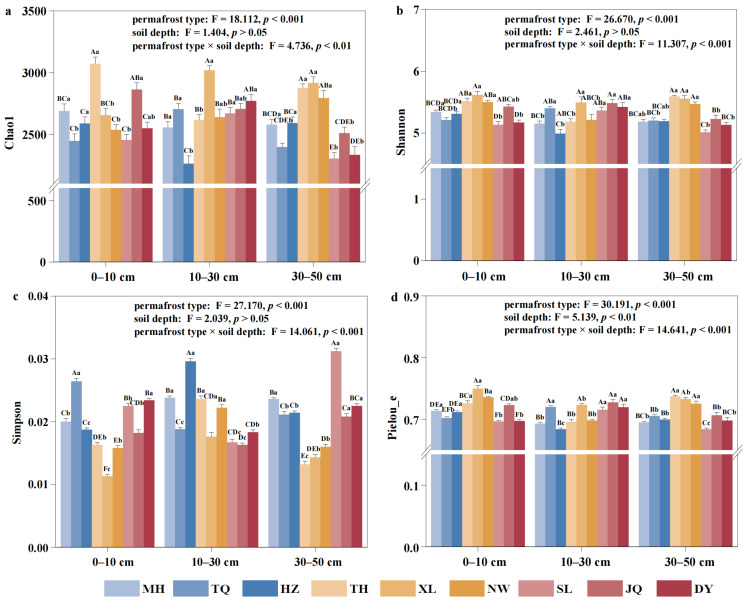
Differences in (**a**) Chao 1, (**b**) Shannon, (**c**) Simpson, and (**d**) Pielou_e indices of bacterial communities in different permafrost peatlands. Different capital letters indicate significant differences among different permafrost peatlands at the same depth (*p* < 0.05); different lowercase letters indicate significant differences at different depths in the same permafrost peatland (*p* < 0.05). MH (Mohe), TQ (Tuqiang), and HZ (Huzhong) are in the continuous permafrost zone. TH (Tahe), XL (Xinlin), and NW (Nanwenghe) are in the discontinuous permafrost zone. SL (Songling), JQ (Jiagedaqi), and DY (Dayangshu) are in the isolated permafrost zone.

Bacterial alpha diversity was higher in the discontinuous permafrost zone than in both the continuous and isolated zones. The Chao1 index was highest at 0–10 cm in Tahe (TH) of the discontinuous permafrost zone (3068.90), and lowest at 10–30 cm in Huzhong (HZ) of the continuous permafrost zone (2260.79, Figure 1a). The maximum Shannon index (5.62) occurred at 0–10 cm in Xinlin (XL) of the discontinuous permafrost zone, whereas the minimum was found at 10–30 cm in HZ of the continuous permafrost zone (4.99, Figure 1b). In contrast, the Simpson index was lowest at 0–10 cm in XL (0.011) and highest at 30–50 cm in Songling (SL) (0.031), which is located in the isolated permafrost zone (Figure 1c). The trend of Pielou_e index was the same as that of the Shannon index (Figure 1d). Two-way ANOVA revealed that permafrost type was the primary driver of bacterial alpha diversity (*p* < 0.001), while soil depth exerted a relatively limited influence, significantly affecting only the Pielou_e index (*p* < 0.01). The interaction between permafrost type and soil depth also significantly influenced bacterial community alpha diversity (*p* < 0.01).

**Figure 2:**
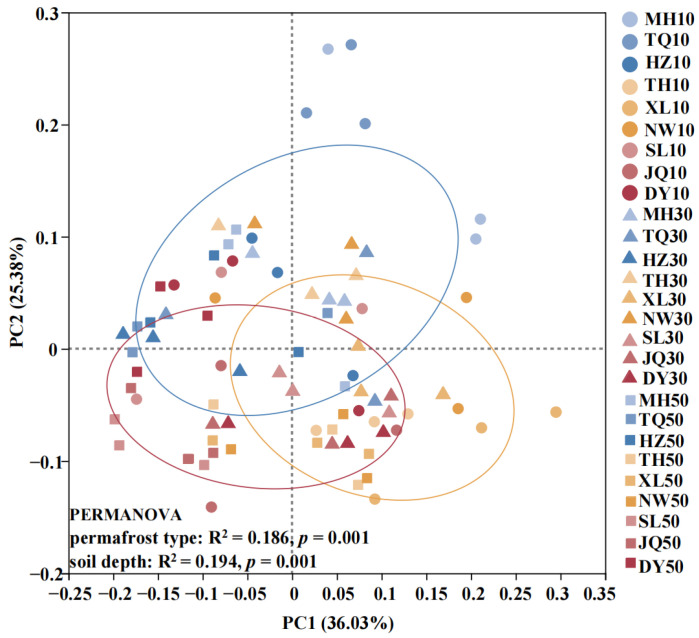
Principal coordinate analysis (PCoA) of soil bacterial community. MH (Mohe), TQ (Tuqiang), and HZ (Huzhong) are in the continuous permafrost zone. TH (Tahe), XL (Xinlin), and NW (Nanwenghe) are in the discontinuous permafrost zone. SL (Songling), JQ (Jiagedaqi), and DY (Dayangshu) are in the isolated permafrost zone. 10: 0–10 cm, 30: 10–30 cm, 50: 30–50 cm.

PCoA based on Bray-Curtis distances revealed that PC1 and PC2 accounted for 36.03% and 25.38% of the total variance, respectively, cumulatively explaining 61.41% (Figure 2). Samples from different permafrost types showed clear spatial clustering: samples from the continuous permafrost zone were mainly distributed in the first and second quadrants, those from the discontinuous permafrost zone were concentrated in the fourth quadrant, and those from the isolated permafrost zone were distributed in the third quadrant. PERMANOVA further confirmed that both permafrost type (R^2^ = 0.186, *p* = 0.001) and soil depth (R^2^ = 0.194, *p* = 0.001) significantly influenced bacterial community composition.

### 3.2. Composition of the Soil Bacterial Communities

**Figure 3:**
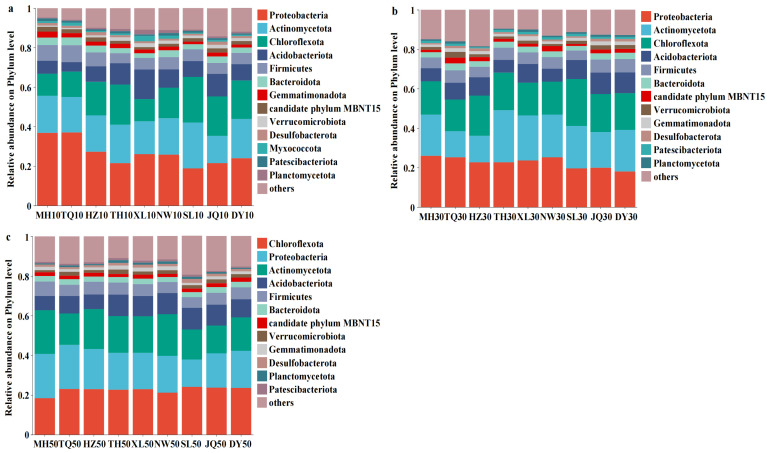
Soil bacterial community composition at phylum level at (**a**) 0–10 cm, (**b**) 10–30 cm and (**c**) 30–50 cm. MH (Mohe), TQ (Tuqiang), and HZ (Huzhong) are in the continuous permafrost zone. TH (Tahe), XL (Xinlin), and NW (Nanwenghe) are in the discontinuous permafrost zone. SL (Songling), JQ (Jiagedaqi), and DY (Dayangshu) are in the isolated permafrost zone.

At the phylum level, Proteobacteria, Actinomycetota, Chloroflexota, Acidobacteriota, and Firmicutes were the dominant taxa (relative abundance > 5%), collectively accounting for over 70% of the total sequences (Figure 3). Along the gradient from the continuous to discontinuous to isolated permafrost zone, the relative abundances of Proteobacteria and Firmicutes showed a gradual decline, whereas Chloroflexota exhibited a consistent increase. Acidobacteriota showed higher relative abundance at 0–10 cm in XL of the discontinuous permafrost zone than in the other plots. The relative abundance of Actinomycetota at 10–30 cm in the discontinuous permafrost zone was higher than that in the continuous and isolated permafrost zones, with the highest value observed at 0–10 cm in SL of the isolated permafrost zone. Across all permafrost types, the relative abundance of Proteobacteria declined with increasing soil depth, while Chloroflexota followed the opposite trend. Acidobacteriota displayed no significant differences among different permafrost types or soil depths.

**Figure 4:**
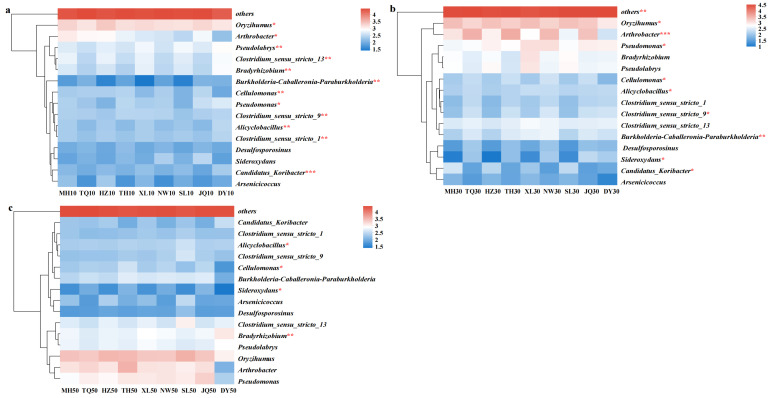
Differences in relative abundance of bacterial community at genus level at (**a**) 0–10 cm, (**b**) 10–30 cm and (**c**) 30–50 cm. Asterisks denote significance levels: * *p* < 0.05, ** *p* < 0.01, *** *p* < 0.001. MH (Mohe), TQ (Tuqiang), and HZ (Huzhong) are in the continuous permafrost zone. TH (Tahe), XL (Xinlin), and NW (Nanwenghe) are in the discontinuous permafrost zone. SL (Songling), JQ (Jiagedaqi), and DY (Dayangshu) are in the isolated permafrost zone.

At the genus level, the dominant genera were *Oryzihumus*, *Arthrobacter*, and *Pseudomonas* (Figure 4). The relative abundances of *Oryzihumus* and *Arthrobacter* gradually decreased along the gradient from the continuous to discontinuous to isolated permafrost zone. At 0–10 cm, the relative abundance of *Arthrobacter* were significantly more abundant in continuous permafrost than in discontinuous and isolated permafrost zones, whereas *Pseudomonas* was significantly less abundant in continuous permafrost than in the other two zones (*p* < 0.05, Figure 4a). At 10–30 cm, *Pseudomonas* displayed higher relative abundance in the discontinuous permafrost zone compared with the continuous and isolated permafrost zones (*p* < 0.05, Figure 4b). At 30–50 cm, *Clostridium_sensu_stricto_13* and *Bradyrhizobium* exhibited increasing abundances along the permafrost gradient, while *Arthrobacter* showed the reverse pattern (Figure 4c).

### 3.3. Co-Occurrence Network of Bacteria

**Figure 5:**
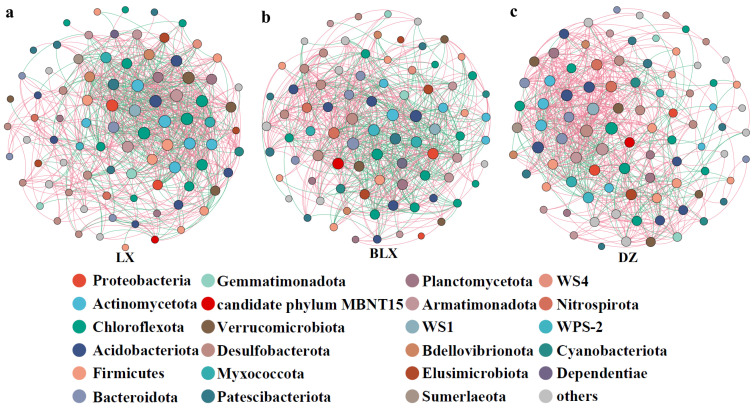
Co-occurrence networks of soil bacterial community in the (**a**) continuous (LX), (**b**) discontinuous (BLX), and (**c**) isolated (DZ) permafrost zones. The color of the nodes represents different bacterial taxa; the size of the nodes is proportional to the number of connections; the lines represent correlations between bacterial taxa, with red lines indicating positive edges and green lines indicating negative edges.

Co-occurrence network analysis revealed that the continuous permafrost zone had the most complex community interactions, with the highest number of total edges (832), average degree (19.126), and network density (0.222, Figure 5a, Table 1), followed by the discontinuous permafrost zone with total edges (775), average degree (18.235), network density (0.217), presenting less complexity (Figure 5b), and the isolated permafrost zone exhibited the lowest network complexity, with total edges (708), average degree (15.910), network density (0.181) (Figure 5c). Negative edges had a higher proportion in the continuous permafrost zone (40.62%) and the discontinuous permafrost zone networks (41.03%), while positive edges predominated in the isolated permafrost zone network (71.61%).

### 3.4. Community Assembly Processes

**Figure 6:**
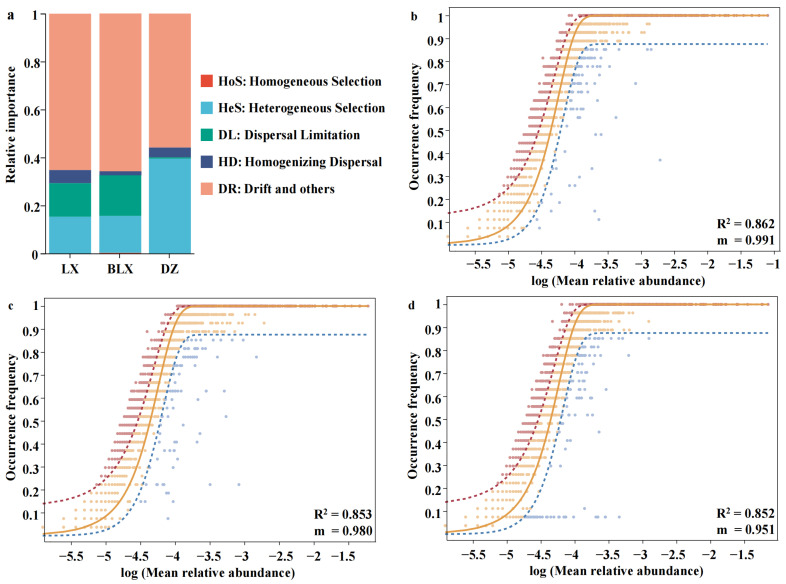
Null model analysis of beta nearest-taxon index (βNTI) for soil bacterial community (**a**); and neutral community model (NCM) fitting for bacterial community (**b**) in the continuous (LX), (**c**) discontinuous (BLX), and (**d**) isolated (DZ) permafrost zones. The solid orange line represents the best fitting trend; the red and blue dashed lines represent the 95% confidence intervals of the prediction model; red dots represent OTUs above the confidence interval; yellow dots represent OTUs within the confidence interval; blue dots represent OTUs below the confidence interval. R^2^ represents the overall goodness of fit of the prediction model and m represents the migration rate of the bacterial community.

βNTI null model analysis indicated that along the gradient from continuous to isolated permafrost zone, the bacterial community relative contribution of heterogeneous selection increased, while that of dispersal limitation decreased (Figure 6a). The NCM further supported this pattern, with the goodness-of-fit (R^2^) and the migration rate (m) declining from 0.862 to 0.852 and 0.991 to 0.951, respectively, from continuous to isolated permafrost zones (Figure 6b–d). These results collectively revealed a gradual shift from stochastic to deterministic community assembly along the permafrost gradient.

### 3.5. Effects of Soil Physicochemical Properties on Bacterial Communities

In the continuous permafrost zone, SWC increased with depth, whereas the opposite trend was observed in the isolated permafrost zone (Table 2). All soil samples were acidic, with pH ranging from 4.39 in XL to 5.45 in Tuqiang (TQ). The contents of TOC and DOC were highest in the continuous permafrost zone and lowest in the isolated permafrost zone, with both showing a significant decreasing trend with depth (except TOC content in TH at 10–30 cm, *p* < 0.05, Table S1). Conversely, TN content was highest at 30–50 cm in SL of the isolated permafrost zone (18.31 g/kg). The highest NH_4_^+^-N content was found at 0–10 cm in TH of the discontinuous permafrost zone (68.48 mg/kg), while the maximum NO_3_^−^-N content occurred at the same depth in Mohe (MH) of the continuous permafrost zone (5.76 mg/kg). TP content varied between 1.38 and 3.67 g/kg, with the lowest value found at 10–30 cm in SL of the isolated permafrost zone, and the highest at 0–10 cm in XL of the discontinuous permafrost zone.

**Figure 7:**
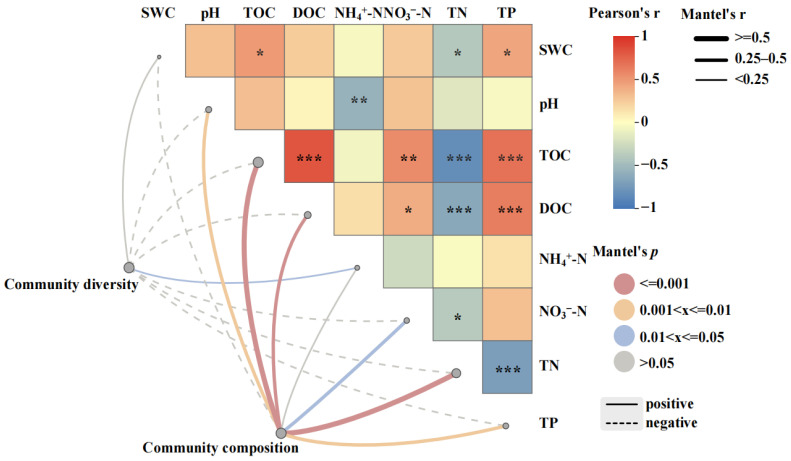
Correlation between soil physicochemical properties and bacterial community composition and diversity. The color of the line indicates significance, the width indicates the strength of the correlation, solid lines represent positive correlations, and dashed lines represent negative correlations. * *p* < 0.05, ** *p* < 0.01, *** *p* < 0.001. SWC: soil water content; TOC: total organic carbon; DOC: dissolved organic carbon; NH_4_^+^-N: ammonium nitrogen; NO_3_^−^-N: nitrate nitrogen; TN: total nitrogen; TP: total phosphorus.

Mantel tests analysis showed that bacterial community composition exhibited highly significant positive correlations with pH, TOC, DOC, TN, TP, and NO_3_^−^-N (*p* < 0.05, Figure 7). Bacterial community diversity was positively correlated only with NH_4_^+^-N (*p* < 0.05).

**Figure 8:**
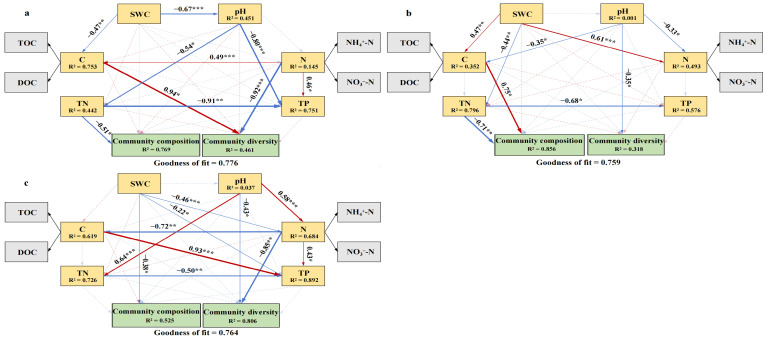
Partial least squares path modeling (PLS-PM) of soil physicochemical properties impacts on bacterial community composition and diversity in the (**a**) continuous (LX), (**b**) discontinuous (BLX), and (**c**) isolated (DZ) permafrost zones. Goodness of fit represents the model fit, solid red arrows represent significant positive correlations, solid blue arrows represent significant negative correlations, dashed red arrows represent non-significant positive correlations, and dashed blue arrows represent non-significant negative correlations. * *p* < 0.05, ** *p* < 0.01, *** *p* < 0.001.

Partial least squares path modeling (PLS-PM) analysis further elucidated the key environmental factors regulating bacterial communities and their pathways of influence in different permafrost zones. In the continuous permafrost zone, TN (coefficients = −0.51, *p* < 0.05) and nitrogen fractions (NH_4_^+^-N and NO_3_^−^-N, coefficients = −0.92, *p* < 0.001) exerted negative direct effects on community composition and diversity, respectively (Figure 8a). SWC indirectly influenced TN by mediating pH, which in turn affected community composition. In the discontinuous permafrost zone, carbon fractions (TOC and DOC) had a positive direct effect on community composition (coefficients = 0.75, *p* < 0.05), whereas TN exerted a negative direct effect on community composition (coefficients = −0.71, *p* < 0.01, Figure 8b). pH showed a negative direct effect on diversity (coefficients = −0.35, *p* < 0.05), while SWC and pH indirectly influenced community composition by regulating carbon fractions. In the isolated permafrost zone, SWC directly and negatively affected community composition (coefficients = −0.38, *p* < 0.05, Figure 8c). pH positively regulated nitrogen fractions, which subsequently negatively impacted diversity.

## 4. Discussion

### 4.1. Bacterial Community Diversity Spatial and Vertical Differentiation

This study revealed that the alpha diversity of bacterial communities first increased and then decreased along the gradient from the continuous to the isolated permafrost zone. O’Brien et al. observed a general decline in prokaryotic alpha diversity following permafrost thaw in three permafrost types in Alaska, and suggested that the decline may be attributed to the enhanced competitive dominance of certain taxa responding to thaw [23]. Wang et al. also found that bacterial alpha diversity decreased with permafrost degradation in alpine meadows of the Qilian Mountains [32]. However, the highest alpha diversity was observed in the discontinuous permafrost zone, implying that the impact of permafrost degradation on microbial diversity does not follow a simple linear framework, but rather results from the combined influence of regional environmental background, thaw progression, and local hydrological conditions. In the continuous permafrost zone of the Greater Khingan Mountains, microbial communities are subjected to persistent dual constraints of low temperature and oligotrophic conditions, allowing only a limited number of cold-tolerant taxa to survive, thereby maintaining diversity at a relatively low level. The discontinuous permafrost zone, as a transitional stage of permafrost degradation, experiences localized warming and the release of previously frozen organic matter, which together provide suitable ecological niches and available substrates for more bacterial taxa, sustaining elevated diversity. Conversely, extensive permafrost loss in the isolated permafrost zone leads to enhanced drainage and habitat fragmentation, resulting in a further decline in diversity. Bacterial community richness and diversity indices were higher at 0–10 or 10–30 cm compared with those at 30–50 cm, aligning with findings from Arctic tundra and Siberian peatlands [33,34]. Notably, the highest diversity in the isolated permafrost zone occurred at 10–30 cm rather than at 0–10 cm, possibly due to enhanced surface drainage caused by active layer thickening. Our results suggest that continued climate warming may drive the depth of maximum genetic diversity downward with active layer deepening, but this requires further testing to confirm the causal relationship between active layer dynamics and the vertical distribution of microbial diversity. Moreover, PCoA and PERMANOVA analyses demonstrated that both permafrost type and soil depth remarkably influenced bacterial community composition. However, with respect to alpha diversity, soil depth only exhibited a significant effect on the Pielou_e index. This differential response indicated that community composition is more sensitive to soil depth than alpha diversity, with the vertical differentiation likely driven by the concurrent decline in substrate and oxygen availability along the soil profile [35].

### 4.2. Spatial Differentiation and Assembly Mechanisms of Bacterial Communities

At the phylum level, Proteobacteria, Actinomycetota, Chloroflexota, Acidobacteriota, and Firmicutes were dominant taxa in all permafrost peatlands, consistent with studies from Alaska, the Arctic, and the Tibetan Plateau, highlighting the widespread adaptability of these bacterial phyla to permafrost habitats [5,36,37]. However, the vertical and spatial variations in their relative abundances reflected differential patterns of habitat filtering and resource competition under different permafrost conditions. Along the gradient from continuous to isolated permafrost, the relative abundance of Proteobacteria showed a steady decreasing trend, whereas that of Chloroflexota exhibited the opposite trend. As typical copiotrophic taxa, Proteobacteria prefer readily available carbon sources, while Chloroflexota, as oligotrophic taxa, are capable of utilizing humic substances [38]. Studies have confirmed that permafrost thaw promotes a shift of dissolved organic matter from high to low aromaticity and molecular weight, accompanied by rapid consumption of labile carbon and transformation of residual organic matter into humic substances [39]. This progressively diminishes the competitive advantage of copiotrophic taxa, explaining the opposing trends of Proteobacteria and Chloroflexota along the permafrost gradient. Additionally, the enrichment of Actinomycetota at 10–30 cm in the discontinuous permafrost zone may be linked to the dynamic balance between released frozen organic matter and mineral-associated carbon protection, in line with observations from Svalbard, where Acidobacteriota dominated the surface layer while Actinomycetota and Chloroflexota prevailed in deeper layers [40]. Co-occurrence network analysis revealed that network complexity decreased successively from the continuous to the isolated permafrost zone, consistent with studies from the Tibetan Plateau and the Alps [26,41]. Complex networks with high connectivity generally possess broader ecological niches and higher resource transfer efficiency, which confer greater resistance to environmental disturbances on the community. The reduction in network complexity weakens the buffering capacity of peatland ecosystems against external disturbances, making previously frozen organic carbon more vulnerable to microbial decomposition into CO_2_ and CH_4_ [42]. Warming experiments have demonstrated that reduced network complexity is closely linked to elevated soil carbon mineralization rates [43]. We speculate that the simplification of microbial interactions and the decline in network stability may indicate an increased risk of organic carbon decomposition in permafrost peatlands of the Greater Khingan Mountains, although direct evidence linking network changes to greenhouse gas emissions requires further investigation. Analyses based on the βNTI null model and the NCM revealed that bacterial community assembly gradually shifted from stochastic to deterministic processes from the continuous to the isolated permafrost zone, a transition likely closely associated with habitat fragmentation and increased environmental heterogeneity regulated by permafrost degradation [44]. In the continuous permafrost zone, where the soil environment is relatively homogeneous, dispersal limitation plays a major role in community assembly. However, in the isolated permafrost zone, the increased frequency of freeze-thaw cycles and the intermingling of permafrost patches with thawed areas amplify spatial heterogeneity in resources and local temperature gradients, subjecting different microbial communities to differential selective pressures and gradually replacing stochastic processes with environmental filtering. This result aligns with the conclusion reported by Thurston et al. from their investigation across six permafrost sites in Alaska, which demonstrated that deterministic processes dominate community assembly following permafrost thaw [45].

### 4.3. Effects of Environmental Factors on Bacterial Communities

Mantel test and PLS-PM analyses indicated that bacterial community composition was jointly regulated by carbon, nitrogen, SWC, and pH, whereas diversity was primarily negatively influenced by nitrogen fractions and pH, with the driving patterns shifting systematically across permafrost types. TN and nitrogen fractions exerted negative effects on both community composition and diversity in the continuous permafrost zone, while carbon fractions positively affected composition but nitrogen fractions showed negative effects in the discontinuous permafrost zone. In the isolated permafrost zone, SWC served as the dominant controlling factor, indicating a staged shift in resource limitation along the permafrost gradient. Restricted by low temperatures in the continuous and discontinuous permafrost zones, soil carbon is mainly derived from ancient carbon pools and vegetation inputs, making carbon the key limiting resource. In the isolated permafrost zone, where permafrost has nearly disappeared, the ancient carbon pool has been largely decomposed or migrated, and the current carbon source is mainly derived from modern vegetation inputs, thereby alleviating carbon limitation and allowing SWC and pH to become the key regulating factors [46]. This shift is consistent with the long-term monitoring results of Kendrick et al. in Alaska, which showed that permafrost thaw led to a fundamental shift in the relative stoichiometric supply of carbon, nitrogen, and phosphorus [47]. These results further suggested that ongoing permafrost degradation may profoundly affect peatland microbial community stability and the turnover of carbon and nitrogen by altering elemental availability and hydrological regimes.

## 5. Conclusions

This study systematically clarified the spatial differentiation patterns and community assembly mechanisms of soil bacterial communities in peatlands across the continuous, the discontinuous, and the isolated permafrost zones in the Greater Khingan Mountains. Bacterial alpha diversity exhibited a trend of first increasing and then decreasing along the permafrost gradient, with the highest value occurring in the discontinuous permafrost zone, accompanied by a transition in dominant taxa from the copiotrophic phylum Proteobacteria to the oligotrophic phylum Chloroflexota. From the continuous to the isolated permafrost zone, bacterial co-occurrence network stability decreased progressively, while community assembly shifted from stochastic to deterministic processes. Concurrently, the primary environmental drivers changed from carbon and nitrogen in the continuous permafrost zone to SWC and pH in the isolated permafrost zone. Our findings provide model parameters specific to each permafrost type for microbial community assembly and network stability, which can improve projections of permafrost peatland carbon dynamics under warming.

## Figures and Tables

**Table 1:** Network properties of soil bacterial community in different permafrost zones.

	Positive Edges	Negative Edges	Total Edges	Average Degree	Netwok Density	Average Clustering Coefficient	Average Path Length
Continuous permafrost zone (LX)	494 (59.38%)	338 (40.62%)	832	19.126	0.222	0.629	2.095
Discontinuous permafrost zone (BLX)	457 (58.97%)	318 (41.03%)	775	18.235	0.217	0.607	2.089
Isolated permafrost zone (DZ)	507 (71.61%)	201 (28.39%)	708	15.910	0.181	0.528	2.263

**Table 2:** Soil physicochemical properties in different types and depths of permafrost zones.

Sample	SWC (%)	pH	TOC (g/kg)	DOC (mg/kg)	NH_4_^+^-N (mg/kg)	NO_3_^−^-N (mg/kg)	TN (g/kg)	TP (g/kg)
MH10	65.38 ± 1.22	5.26 ± 0.03	418.28 ± 5.39	289.67 ± 3.33	12.56 ± 0.45	5.76 ± 0.03	8.36 ± 0.19	3.36 ± 0.02
MH30	68.17 ± 1.29	5.23 ± 0.02	346.85 ± 5.07	207.43 ± 2.88	18.96 ± 0.59	3.69 ± 0.01	10.47 ± 0.29	3.13 ± 0.03
MH50	70.33 ± 1.45	5.18 ± 0.03	300.12 ± 4.82	155.78 ± 2.52	15.29 ± 0.61	2.77 ± 0.03	11.45 ± 0.40	2.27 ± 0.02
TQ10	61.84 ± 1.13	5.45 ± 0.05	433.62 ± 5.50	271.85 ± 3.10	14.69 ± 0.47	4.57 ± 0.05	9.36 ± 0.33	2.08 ± 0.05
TQ30	66.31 ± 1.33	5.11 ± 0.02	342.04 ± 5.03	221.20 ± 2.75	11.21 ± 0.52	3.64 ± 0.04	11.98 ± 0.41	2.55 ± 0.04
TQ50	69.76 ± 1.40	5.15 ± 0.03	293.72 ± 4.60	168.05 ± 2.17	8.95 ± 0.39	5.03 ± 0.05	17.05 ± 0.48	1.94 ± 0.03
HZ10	67.96 ± 1.26	5.03 ± 0.01	391.52 ± 5.08	243.01 ± 2.83	26.78 ± 0.72	3.96 ± 0.02	12.10 ± 0.39	3.59 ± 0.02
HZ30	70.11 ± 1.41	4.86 ± 0.02	312.31 ± 4.77	204.62 ± 2.46	23.15 ± 0.79	4.75 ± 0.01	14.87 ± 0.40	3.08 ± 0.05
HZ50	71.23 ± 1.48	5.01 ± 0.03	268.41 ± 4.52	155.16 ± 2.30	19.85 ± 0.68	3.02 ± 0.04	16.03 ± 0.43	2.11 ± 0.04
TH10	60.21 ± 1.14	4.56 ± 0.04	320.92 ± 4.70	251.11 ± 2.87	68.48 ± 1.71	3.44 ± 0.02	13.96 ± 0.25	2.86 ± 0.04
TH30	66.81 ± 1.43	5.18 ± 0.03	309.14 ± 4.61	230.78 ± 2.76	60.17 ± 1.56	3.59 ± 0.02	13.05 ± 0.22	3.03 ± 0.05
TH50	62.58 ± 1.15	4.85 ± 0.02	241.16 ± 4.42	196.75 ± 2.32	43.96 ± 1.48	3.01 ± 0.05	15.21 ± 0.28	1.49 ± 0.03
XL10	68.41 ± 1.18	4.39 ± 0.04	367.42 ± 5.07	241.03 ± 2.50	50.39 ± 1.15	4.47 ± 0.03	8.79 ± 0.15	3.67 ± 0.03
XL30	70.05 ± 1.52	4.97 ± 0.05	323.73 ± 4.93	202.51 ± 2.29	43.31 ± 1.21	3.91 ± 0.05	11.35 ± 0.17	3.02 ± 0.04
XL50	62.36 ± 1.13	4.83 ± 0.05	247.33 ± 4.78	167.19 ± 2.32	39.96 ± 1.02	2.86 ± 0.05	14.96 ± 0.39	2.47 ± 0.05
NW10	71.94 ± 1.43	4.67 ± 0.03	358.91 ± 5.19	237.91 ± 2.71	61.73 ± 1.42	4.52 ± 0.01	11.08 ± 0.16	2.56 ± 0.04
NW30	64.95 ± 1.32	5.12 ± 0.04	331.01 ± 4.65	196.88 ± 2.42	50.23 ± 1.28	3.39 ± 0.02	12.66 ± 0.21	2.22 ± 0.03
NW50	63.87 ± 1.36	4.81 ± 0.02	255.82 ± 4.30	170.03 ± 2.19	54.61 ± 1.35	2.18 ± 0.01	15.02 ± 0.28	2.50 ± 0.02
SL10	64.35 ± 1.26	4.71 ± 0.05	288.97 ± 4.54	258.71 ± 2.80	32.60 ± 0.97	2.85 ± 0.03	16.25 ± 0.36	2.29 ± 0.03
SL30	61.79 ± 1.20	4.88 ± 0.04	259.25 ± 4.40	179.83 ± 2.28	40.25 ± 0.98	3.39 ± 0.05	17.58 ± 0.34	1.38 ± 0.04
SL50	58.33 ± 1.07	5.03 ± 0.04	185.72 ± 4.08	153.25 ± 1.78	21.86 ± 0.68	3.76 ± 0.03	18.31 ± 0.41	1.44 ± 0.02
JQ10	63.92 ± 1.21	4.45 ± 0.02	315.21 ± 4.61	233.51 ± 2.71	39.58 ± 1.14	2.68 ± 0.01	14.28 ± 0.34	2.37 ± 0.04
JQ30	59.03 ± 1.14	4.52 ± 0.03	267.37 ± 4.23	160.22 ± 2.39	44.28 ± 0.91	4.29 ± 0.02	13.04 ± 0.27	1.83 ± 0.05
JQ50	55.78 ± 1.03	4.79 ± 0.02	219.58 ± 4.28	137.85 ± 2.17	28.76 ± 0.59	3.38 ± 0.04	18.05 ± 0.38	1.52 ± 0.03
DY10	56.99 ± 1.07	4.73 ± 0.02	331.23 ± 5.02	245.75 ± 2.67	42.14 ± 1.35	3.80 ± 0.05	14.01 ± 0.28	2.85 ± 0.02
DY30	53.68 ± 1.01	4.50 ± 0.05	251.85 ± 4.11	189.03 ± 2.38	30.25 ± 0.80	2.67 ± 0.05	12.12 ± 0.23	2.69 ± 0.04
DY50	49.85 ± 0.97	4.87 ± 0.03	224.10 ± 4.44	144.74 ± 2.25	23.33 ± 0.73	3.53 ± 0.03	17.87 ± 0.28	1.71 ± 0.05

Note: Data are presented as mean ± standard error (*n* = 3). MH (Mohe), TQ (Tuqiang), and HZ (Huzhong) are in the continuous permafrost zone. TH (Tahe), XL (Xinlin), and NW (Nanwenghe) are in the discontinuous permafrost zone. SL (Songling), JQ (Jiagedaqi), and DY (Dayangshu) are in the isolated permafrost zone. 10: 0–10 cm, 30: 10–30 cm, 50: 30–50 cm.

## Data Availability

The data presented in this study are available on request from the corresponding author.

## Supplementary Materials

The following supporting information can be downloaded at https://www.mdpi.com/article/10.3390/microorganisms14071558/s1, Table S1: Soil physicochemical properties in different types and depths of permafrost zones.
