# Supplementary material for: Spatial Differentiation and Community Assembly of Soil Bacterial Communities in Permafrost Peatlands of the Greater Khingan Mountains
Source: Microorganisms. 2026 Jul 16;14(7):1558. doi: 10.3390/microorganisms14071558 (PMC13413961; doi:10.3390/microorganisms14071558)
Supplement: Supplementary file 1 [file microorganisms-14-01558-s001.zip › microorganisms-4419912-supplementary.pdf]

Table S1. Soil physicochemical properties in different types and depths of permafrost zones.

Sample	SWC (%)	pH	TOC (g/kg)	DOC (mg/kg)	NH_4^+-N (mg/kg)	NO_3^--N (mg/kg)	TN (g/kg)	TP (g/kg)
MH10	65.38±1.22BCa	5.26±0.03Ba	418.28±5.39Aa	289.67±3.33Aa	12.56±0.45Gc	5.76±0.03Aa	8.36±0.19Db	3.36±0.02Ba
MH30	68.17±1.29ABa	5.23±0.02Aa	346.85±5.07Ab	207.43±2.88Bb	18.96±0.59Ea	3.69±0.01Db	10.47±0.29Ea	3.13±0.03Ab
MH50	70.33±1.45Aa	5.18±0.03Aa	300.12±4.82Ac	155.78±2.52Cc	15.29±0.61Eb	2.77±0.03Ec	11.45±0.40Ea	2.27±0.02Bc
TQ10	61.84±1.13CDb	5.45±0.05Aa	433.62±5.50Aa	271.85±3.10Ba	14.69±0.47Ga	4.57±0.05Bb	9.36±0.33Dc	2.08±0.05Fb
TQ30	66.31±1.33ABab	5.11±0.02ABb	342.04±5.03ABb	221.20±2.75Ab	11.21±0.52Fb	3.64±0.04Dc	11.98±0.41CDb	2.55±0.04Ba
TQ50	69.76±1.40ABa	5.15±0.03ABb	293.72±4.60Ac	168.05±2.17Bc	8.95±0.39Fc	5.03±0.05Aa	17.05±0.48ABCa	1.94±0.03Cb
HZ10	67.96±1.26ABa	5.03±0.01Ca	391.52±5.08Ba	243.01±2.83DEa	26.78±0.72Fa	3.96±0.02Cb	12.10±0.39Cb	3.59±0.02Aa
HZ30	70.11±1.41Aa	4.86±0.02Cb	312.31±4.77Cb	204.62±2.46Bb	23.15±0.79Eb	4.75±0.01Aa	14.87±0.40Ba	3.08±0.05Ab
HZ50	71.23±1.48Aa	5.01±0.03BCa	268.41±4.52Bc	155.16±2.30Cc	19.85±0.68Dc	3.02±0.04Dc	16.03±0.43BCDa	2.11±0.04Bc
TH10	60.21±1.14CDb	4.56±0.04EFc	320.92±4.70Da	251.11±2.87CDa	68.48±1.71Aa	3.44±0.02Db	13.96±0.25Bb	2.86±0.04Ca
TH30	66.81±1.43ABa	5.18±0.03Aa	309.14±4.61Ca	230.78±2.76Ab	60.17±1.56Ab	3.59±0.02Da	13.05±0.22Cb	3.03±0.05Aa
TH50	62.58±1.15Cab	4.85±0.02Db	241.16±4.42CDb	196.75±2.32Ac	43.96±1.48Bc	3.01±0.05Dc	15.21±0.28CDa	1.49±0.03Eb
XL10	68.41±1.18ABa	4.39±0.04Gb	367.42±5.07BCa	241.03±2.50DEa	50.39±1.15Ca	4.47±0.03Ba	8.79±0.15Dc	3.67±0.03Aa
XL30	70.05±1.52Aa	4.97±0.05BCa	323.73±4.93BCb	202.51±2.29Bb	43.31±1.21Cb	3.91±0.05Cb	11.35±0.17DEb	3.02±0.04Ab
XL50	62.36±1.13Cb	4.83±0.05Da	247.33±4.78BCc	167.19±2.32Bc	39.96±1.02Bb	2.86±0.05DEc	14.96±0.39Da	2.47±0.05Ac
NW10	71.94±1.43Aa	4.67±0.03DEc	358.91±5.19Ca	237.91±2.71DEa	61.73±1.42Ba	4.52±0.01Ba	11.08±0.16Cc	2.56±0.04Da
NW30	64.95±1.32ABCb	5.12±0.04ABa	331.01±4.65ABCb	196.88±2.42BCb	50.23±1.28Bb	3.39±0.02Eb	12.66±0.21CDb	2.22±0.03Cb
NW50	63.87±1.36BCb	4.81±0.02Db	255.82±4.30BCc	170.03±2.19Bc	54.61±1.35Ab	2.18±0.01Fc	15.02±0.28Da	2.50±0.02Aa
SL10	64.35±1.26BCa	4.71±0.05DEb	288.97±4.54Ea	258.71±2.80BCa	32.60±0.97Eb	2.85±0.03Ec	16.25±0.36Ac	2.29±0.03Ea
SL30	61.79±1.20BCab	4.88±0.04Cab	259.25±4.40Db	179.83±2.28Db	40.25±0.98Ca	3.39±0.05Eb	17.58±0.34Aab	1.38±0.04Eb
SL50	58.33±1.07CDb	5.03±0.04ABa	185.72±4.08Ec	153.25±1.78Cc	21.86±0.68Dc	3.76±0.03Ba	18.31±0.41Aa	1.44±0.02Eb
JQ10	63.92±1.21BCa	4.45±0.02FGb	315.21±4.61Da	233.51±2.71Ea	39.58±1.14Db	2.68±0.01Fc	14.28±0.34Bb	2.37±0.04Ea
JQ30	59.03±1.14CDab	4.52±0.03Db	267.37±4.23Db	160.22±2.39Eb	44.28±0.91Ca	4.29±0.02Ba	13.04±0.27Cb	1.83±0.05Db
JQ50	55.78±1.03DEc	4.79±0.02Da	219.58±4.28Dc	137.85±2.17Dc	28.76±0.59Cc	3.38±0.04Cb	18.05±0.38Aa	1.52±0.03Ec
DY10	56.99±1.07Da	4.73±0.02Da	331.23±5.02Da	245.75±2.67CDEa	42.14±1.35Da	3.80±0.05Ca	14.01±0.28Bb	2.85±0.02Ca
DY30	53.68±1.01Dab	4.50±0.05Db	251.85±4.11Db	189.03±2.38CDb	30.25±0.80Db	2.67±0.05Fb	12.12±0.23CDc	2.69±0.04Bb
DY50	49.85±0.97Eb	4.87±0.03CDb	224.10±4.44Dc	144.74±2.25CDc	23.33±0.73Dc	3.53±0.03Cc	17.87±0.28ABa	1.71±0.05Dc

Note: Data are presented as mean ± standard error (n = 3). Different capital letters indicate significant differences among different types of permafrost zones at the same depth ($p < 0.05$); different lowercase letters indicate significant differences at different depths in the same type of permafrost zone ($p < 0.05$). MH (Mohe), TQ (Tuqiang), and HZ (Huzhong) are in the continuous permafrost zone. TH (Tahe), XL (Xinlin), and NW (Nanwenghe) are in the discontinuous permafrost zone. SL (Songling), JQ (Jiagedaqi), and DY (Dayangshu) are in the isolated permafrost zone. 10: 0–10 cm, 30: 10–30 cm, 50: 30–50 cm.
